# Hemi-hamate Arthroplasty in Chronic Fracture-dislocation of Proximal Interphalangeal Joint of Fingers: A Report of Two Cases

**DOI:** 10.7759/cureus.6735

**Published:** 2020-01-22

**Authors:** Syahril Rizal Arsad, Sei Haw Sem, Jeremy Prakash Silvanathan, Rashdeen Fazwi Muhammad Nawawi

**Affiliations:** 1 Orthopaedics, Hospital Kuala Lumpur, Kuala Lumpur, MYS

**Keywords:** arthroplasty, fracture-dislocation, proximal interphalangeal joint, hemi-hamate

## Abstract

Dorsal dislocations of proximal interphalangeal joint with palmar lip fractures base of middle phalanx of fingers are rare, complex, and often a challenging injury to the treating hand surgeons especially in those chronic cases. Hemi-hamate arthroplasty is the preferred surgical option in treating chronic dorsal fracture-dislocations of the proximal interphalangeal joint. We report two cases with a chronic injury that have been treated with hemi-hamate arthroplasty. Range of motion, pinch and grip strengths, QuickDASH scores, complications, and radiological findings were recorded at follow-up. Good functional outcomes were observed in both patients without major complications. Hemi-hamate arthroplasty can be a reliable surgical treatment for chronic proximal interphalangeal joint fracture-dislocations.

## Introduction

Dorsal fracture-dislocations of finger proximal interphalangeal (PIP) joint are uncommon, difficult to treat and debilitating injuries. Without proper management, it often leads to chronic pain, stiffness, and poor hand function. The mechanism of injury is commonly due to a combination of axial and hyperextension forces through the PIP joint secondary to motor vehicle accident and sports injury [1]. There is no one gold standard treatment in treating dorsal fracture-dislocation of PIP joint. Occasionally, some of the patients presented late for treatment with malunion and chronic subluxation of the PIP joint. Resurfacing of the articular surface is the only option in these patients. We report two cases with chronic fracture-dislocation of PIP joint that treated successfully with hemi-hamate arthroplasty (HHA). 

## Case presentation

Case report one

**Figure 1:**
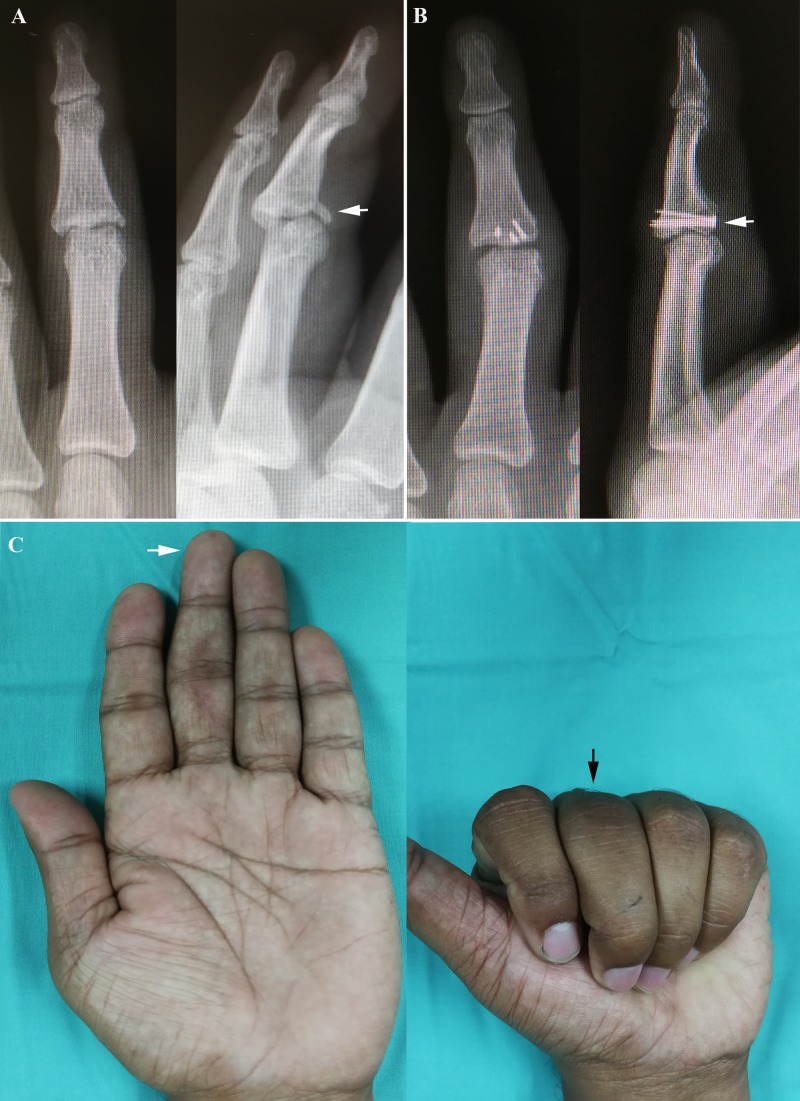
Case Report One (A) Preoperative radiographs showed fracture base of middle phalanx left middle finger with dorsal subluxation of proximal interphalangeal joint (white arrow). (B) Postoperative radiographs revealed graft union with no joint subluxation (white arrow). (C) After 12 months, good functional range of motion of the left middle finger in extension (white arrow) and flexion (black arrow).

A 28-year-old man presented six months after an injury in the futsal game, which was missed initially. He had persistent pain, swelling, and stiffness over the left middle finger (MF) PIP joint. The radiographs showed a fracture base of the proximal phalanx with dorsal subluxation of the PIP joint (Figure 1A). He underwent HHA at 28 weeks after injury. There was no perioperative complication and he underwent a structured postoperative rehabilitation, which involved splinting, range of motion (ROM), and pinch and grip strengthening exercises. On follow-up at 12 months, the radiographs showed a union of the graft with no joint subluxation (Figure 1B). ROM for the distal and PIP joints were 0-80^o^ and 0-75^o ^, respectively (Figure 1C). His grip strength was 20 kg/force (62.5% of normal side) and pinch strength was 4.5 kg/force (90% of normal side). There was no pain over the PIP joint and donor site. He returned to his job as a storekeeper with the QuickDASH score of 4.5. 

Case report two

**Figure 2:**
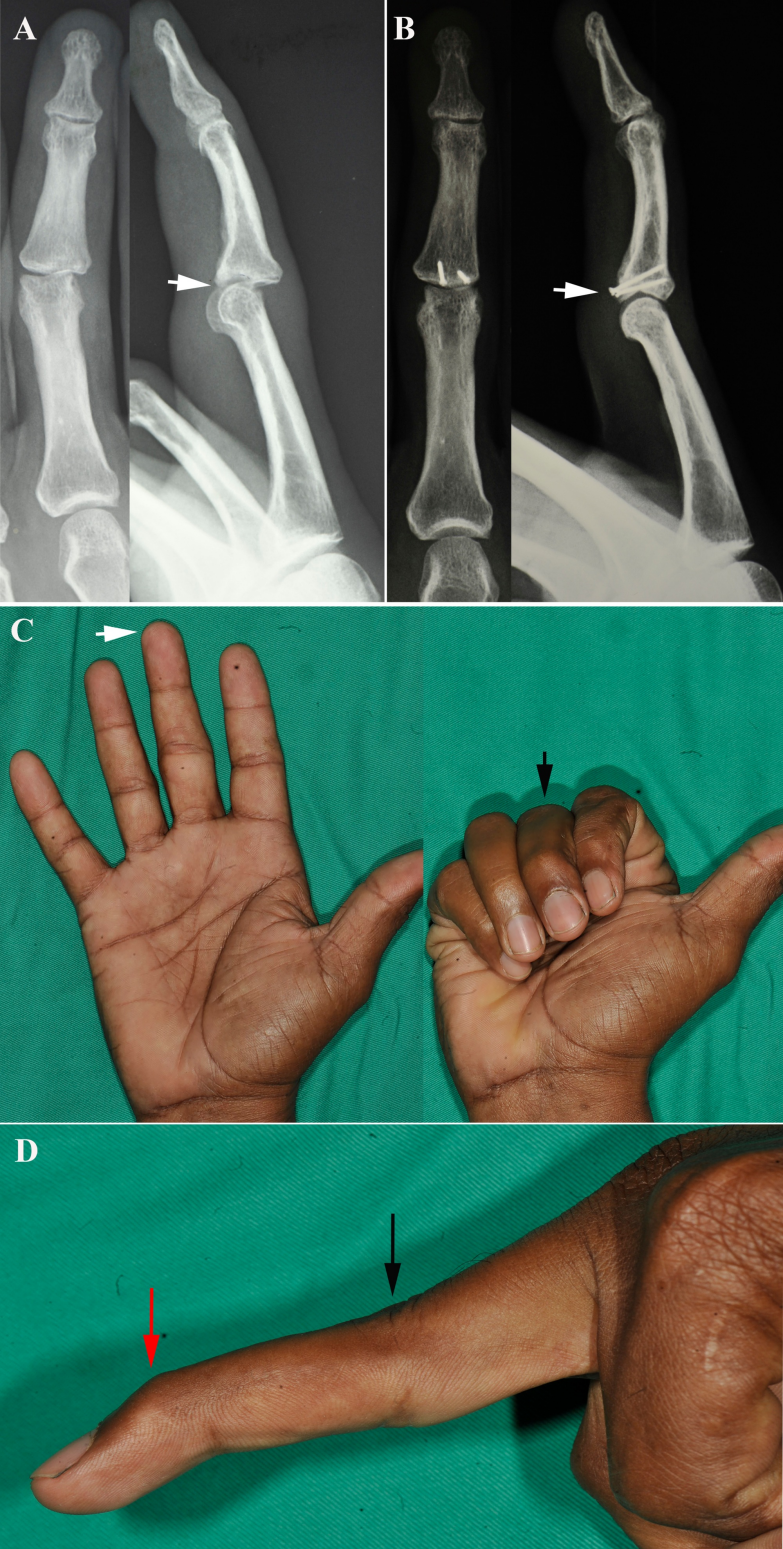
Case Report Two (A) Preoperative radiographs showed malunited base of middle phalanx right middle finger with dorsal subluxation of proximal interphalangeal joint (white arrow). (B) Postoperative radiographs at 24 months follow-up revealed graft union with no joint subluxation (white arrow). (C) At 24 months follow-up, good functional range of motion of the right middle finger in extension (white arrow) and flexion (black arrow) despite swan neck deformity of the finger, where the proximal interphalangeal joint was hyperextended (black arrow) and distal interphalangeal joint was flexed (red arrow) (D).

A 48-year-old man with closed fracture-dislocation of right MF PIP joint after a fall in outstretched hand was referred to our hospital at three months after injury due to failure of conservative treatment (splinting). Radiographs revealed malunion of fracture with dorsal subluxation of the right MF PIP joint (Figure 2A). HHA was performed at 32 weeks after injury. On follow-up at two years, good union of the graft with no joint subluxation was noted from the radiographs (Figure 2B). However, there was swan neck deformity with ROM of PIP joint -10-70^o^ and distal interphalangeal joint 20-50^o^ (Figure 2C, 2D). He regained good grip and pinch strength, which were 32 kg/force (88.9 % of normal side) and 6.0 kg/force (92.3% of normal side), respectively. He returned to his previous job as a lorry driver with the QuickDASH score of 9.1. 

Surgical technique

**Figure 3:**
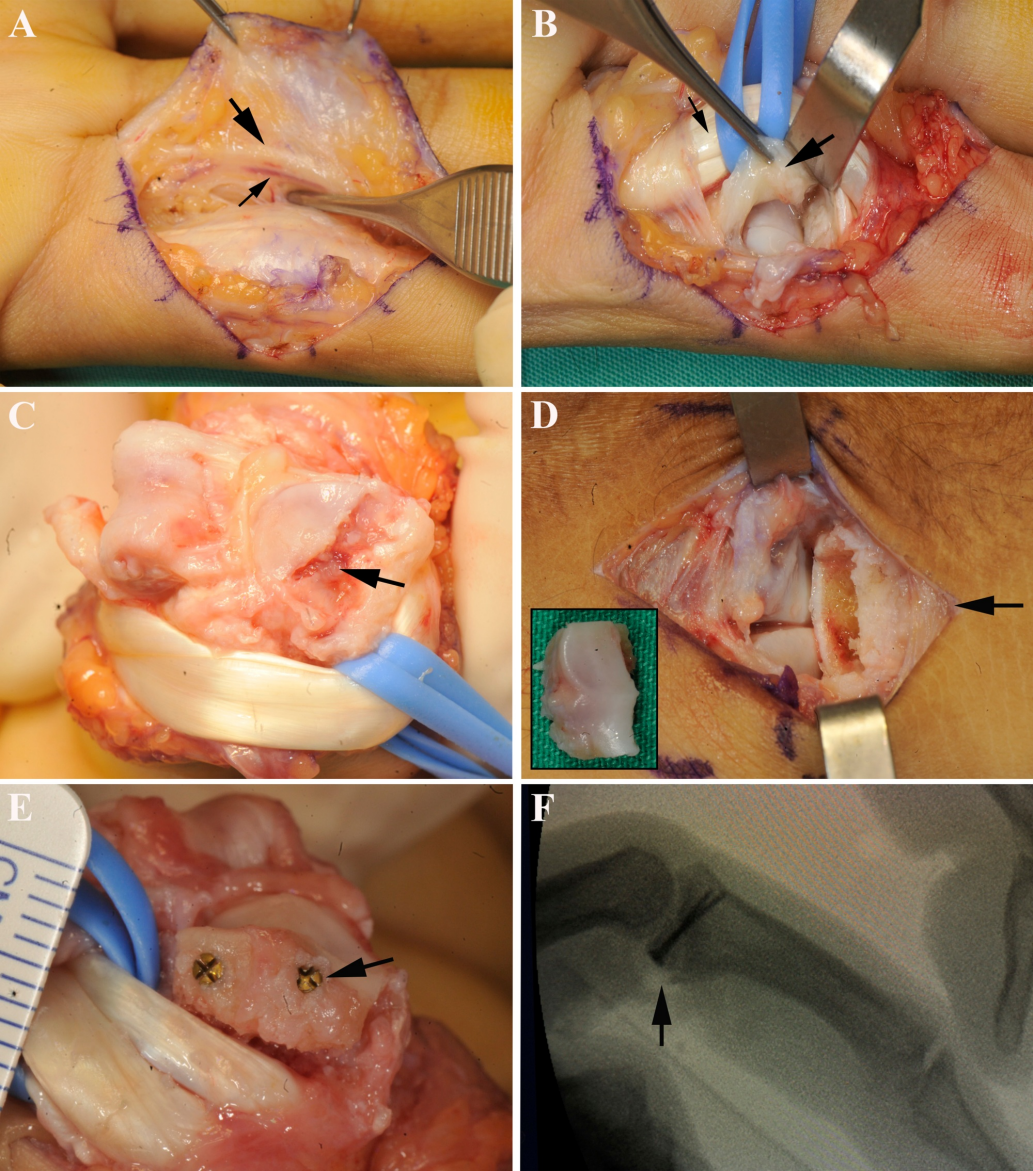
Surgical Technique (A) Volar approach with Bruner skin incision, both the digital nerves (big black arrow) and arteries (small black arrow) were identified and protected. (B) Flexor tendon sheath was incised between A2 and A4 pulleys, flexor tendons (small black arrow) were reflected laterally, and volar plate (big black arrow) was divided distally from the base of middle phalanx and laterally from accessory collateral ligaments. (C) Proximal interphalangeal joint was exposed with the finger hyperextended in a shotgun manner, fracture site (black arrow) was evaluated. (D) Hamate graft (small black box) was harvested through a longitudinal incision over the 4^th^/5^th^ carpometacarpal joint (black arrow). (E) The graft was fixed with two screws size 1.0mm (black arrow). (F) Position of screws and graft were checked with image intensifier (black arrow).

We performed the surgeries with the similar technique described by Williams et al [2]. The PIP joint was accessed through the palmar approach with neurovascular structures identified and mobilized carefully (Figure 3A). The flexor tendon sheath was incised between A2 and A4 pulleys in a Zig-Zag pattern and flexor tendons were reflected laterally to expose the underlying volar plate (VP). The VP was divided distally from the base of the middle phalanx and laterally from accessory collateral ligaments (Figure 3B). PIP joint was exposed with the finger hyperextended in a shotgun manner and fracture site was evaluated, prepared and measured for HHA (Figure 3C). The distal part of the hamate was exposed through a longitudinal incision over the 4^th^/5^th^ carpometacarpal joint with dorsal ulnar sensory branches identified and protected. Adequate size (slightly larger than measured) of the hamate graft was harvested with oscillating saw and osteotome (Figure 3D). The graft was trimmed to match the defect and stabilized with 0.8mm k-wire temporarily before final fixation with two or three screws size 1.0mm (Figure 3E). The joint is reduced, the position of screws and graft were checked with image intensifier before closure (Figure 3F). The flexor sheath and VP were repaired to prevent joint hyperextension. 

## Discussion

The primary aim of treatment for acute dorsal fracture-dislocation of the PIP joint is to achieve a stable and concentric reduction that allows early smooth active motion. The treatment choice is based on the congruity and stability of the joint post closed reduction, and the options include dorsal block splint, distractive dynamic fixator, open reduction and internal fixation, VP arthroplasty, and HHA.

There are not many treatment options available for unstable and chronic dorsal fracture-dislocation of the finger PIP joint. VP arthroplasty is an interposition arthroplasty to restore volar soft tissue buttress by tethering to the dorsally unstable PIP joint and reliably restored function in unstable fracture-dislocation involved less than 50% articular surface [1]. However, the risk of joint redislocation and persistent PIP joint flexion contracture increase as the fracture severity increases (> 50% of articular surface), and poorer outcome when performed for injuries more than six weeks [1-2]. Therefore, HHA is preferable for chronic fracture-dislocation of finger PIP joint. 

Both our patients showed comparable functional outcomes with the published literatures on HHA in chronic fracture-dislocations of finger PIP joint (Table 1) [1,3-4]. However, the grip strength for Case One was lower than the others because it involved the nondominant hand and could be improved with a longer duration of follow-up. The arc of motion of the distal interphalangeal joint for Case Two was less than others mainly because of the swan neck deformity, which could be due to attenuation of volar plate repair. Nonetheless, he has no pain or functional limitations that required surgical correction. 

**Table 1:** Case series on hemi-hamate arthroplasty in patients with chronic fracture-dislocation of proximal interphalangeal joint. DASH - Disabilities of the Arm, Shoulder, and Hand

Publications	No of Patients	Delay to Surgery (Weeks)	Range of Motion Arc (Degrees)		Grip Strength (% of normal side)	Mean Follow-up (Range) (Months)	DASH/QuickDASH Score
			Proximal Interphalangeal Joint	Distal Interphalangeal Joint			
Calfee et al. (2009) [3]	8	30	69	51	82	54 (12 - 87)	9 (DASH)
Lindenblatt et al. (2013) [4]	5	22 (6 - 53)	65	-	-	9 (3 - 14)	-
Burnier et al. (2016) [1]	10	> 6	63	43	87	24 (5 - 73)	14
Our patients	Case 1	28	75	80	62.5	12	4.5
	Case 2	32	80	30	88.9	24	9.1

**Figure 4:**
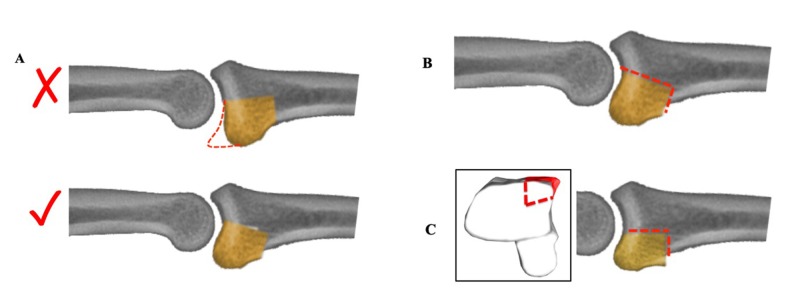
(A) It is important to restore volar buttress to prevent recurrent dorsal subluxation, red dashed line indicates correct position of the graft. (B) A distal volarly directed slope of recipient bed preparation (red dashed line) is useful in positioning the correct angle of the graft articular surface. (C) A proximal directed volarly oblique cutting (red dashed line) in harvesting hemihamate graft in the coronal plane (black box) with a box-shaped recipient bed to ensure adequate tilting of the graft articular surface.

The major challenge for a successful HHA is to reproduce a palmar lip to ensure adequate volar buttress and prevent recurrent dorsal subluxation (Figure 4A). There are two different techniques described to achieve the purpose. First is by preparing the recipient bed with slight palmar slope towards distally (Figure 4B). Second is by harvesting the graft with a volarly oblique cutting in the coronal plane direction [5] (Figure 4C). The basic principle behind both techniques is to redirect the harvested graft with appropriate tilting of the articular surface to restore a cup-shaped geometry for the base of middle phalanx. 

## Conclusions

HHA is a technically challenging procedure that can restore damaged articular surface, recreate joint stability, and allow early ROM exercise in PIP joint with chronic injuries. Proper surgical techniques and a good understanding of the PIP joint anatomy are essential for a successful surgery. In conclusion, HHA is a valuable surgical procedure in treating chronic dorsal fracture-dislocation of finger PIP joint with promising short-term functional outcomes. However, long term follow-up studies are needed to assess the rate of osteoarthritis. 
